# Abstracts and Selections

**Published:** 1912-06

**Authors:** 


### Abstracts and Selections.

THE NATHAN STRAUSS PREVENTORIUM FOR CHILDREN, at Farmingdale, N. J., was dedicated on April 25th. It is the first of its kind in this country. It takes children from 4 to 14 years of age, from homes where tuberculosis exists, or who belong to that class which von Behring aptly classes as "consumption candidates." At the preventorium the children are in the fresh air every minute of the day and night. They sleep on porches entirely open in front, take their meals in a dining room whose large windows are always open, go to school either under the trees, or in a class room open on all sides to the air, and spend their playtime in the open fields and woods. While the child is at the preventorium, a trained social worker visits the home of the parent and recommends changes that may be necessary in order to make the home safe for the child on its return. In this it is assisted by the Department of Health, the Department of Charities, the Association of Tuberculosis Clinics, the ladies' auxiliaries, organized relief agencies, and many others. Some 350 children have already been treated at the preventorium, and the results have been most encouraging.—*Editorial,* May 4th number, *New York Medical Journal.*

---

SEPSIS in most puerperal cases starts as wound infection at the vulva or lower end of the vagina, and can generally be prevented by moistening the vulva pads with bichloride solution 1 to 5000. This should be continued for about five days.

THE umbilical cord should not be cut until after it stops pulsating, especially when the labor has been severe.

A WOMAN should not be starved after delivery. The more food she is given within reason the better.

THE breasts should never be massaged or pumped, as disregard of this caution may lead to mammary abscess.—Waldo, *International Journal of Surgery,* April, 1912.

---

"SCHOOL HYGIENE demands among other things that physical training be recognized as important as mental training; that the causes of overburdening, if they exist, be scientifically fixed and then removed; that the physiological age of a child and not its chronological age should determine its admission into school; that children should be classified as to capabilities; that home work should be regulated; that there be sex enlightenment; that inquiries be made as to the effect of co-education, instruction in the open air," etc., and it may be answered that the underfed should be fed, the naked clothed and the dirty cleansed.—Staubenmüller, *New York Medical Journal,* April, 1912.

---

AS SOLUTIONS OF COCAINE decompose, they should always be freshly prepared; likewise the effectiveness of the solution is increased by warming it at about the body temperature and by adding a few drops of adrenalin chloride.—Morrow, *New York Medical Journal,* February, 1912.

---

"COITUS will not cure masturbation, and is a dangerous experiment.

"When masturbation has been firmly established, you can no more talk your patient out of masturbating than you can talk a child suffering from scabies out of scratching."—Hühner, *New York Medical Journal,* February, 1912.

---

EQUAL parts of castor oil and aromatic syrup of rhubarb constitutes Simon Solis Cohen's "candy medicine" among the children of Philadelphia.—*New York Medical Journal,* February, 1912.

---

Aren't we picking up folks just as fast as they fall?
And shall this man dictate to us? Shall he?
Why should people of sense stop to put up a fence,
While the ambulance works in the valley?"

—Sent by Dr. Bogart.

